# Ki-67 assessment of pancreatic neuroendocrine neoplasms: Systematic review and meta-analysis of manual vs. digital pathology scoring

**DOI:** 10.1038/s41379-022-01055-1

**Published:** 2022-03-05

**Authors:** Claudio Luchini, Liron Pantanowitz, Volkan Adsay, Sylvia L. Asa, Pietro Antonini, Ilaria Girolami, Nicola Veronese, Alessia Nottegar, Sara Cingarlini, Luca Landoni, Lodewijk A. Brosens, Anna V. Verschuur, Paola Mattiolo, Antonio Pea, Andrea Mafficini, Michele Milella, Muhammad K. Niazi, Metin N. Gurcan, Albino Eccher, Ian A. Cree, Aldo Scarpa

**Affiliations:** 1 Department of Diagnostics and Public Health, Section of Pathology, University of Verona, Verona, Italy; 2 ARC-Net Research Center, University and Hospital Trust of Verona, Verona, Italy; 3 Department of Pathology & Clinical Labs, University of Michigan, Ann Arbor, MI USA; 4 Department of Pathology, Koç University Hospital and Koç University Research Center for Translational Medicine (KUTTAM), Istanbul, Turkey; 5 University Hospitals Cleveland Medical Center, Case Western Reserve University, Cleveland, OH USA; 6 Division of Pathology, San Maurizio Central Hospital, Bolzano, Italy; 7 Department of Internal Medicine and Geriatrics, University of Palermo, Palermo, Italy; 8 Pathology Unit, Azienda Ospedaliera Universitaria Integrata (AOUI), Verona, Italy; 9 Department of Medicine, Section of Oncology, University and Hospital Trust of Verona, Verona, Italy; 10 Department of Surgery, The Pancreas Institute, University and Hospital Trust of Verona, Verona, Italy; 11 Department of Pathology, University Medical Center Utrecht, Utrecht, The Netherlands; 12 Center for Biomedical Informatics, Wake Forest School of Medicine, Winston Salem, NC USA; 13 International Agency for Research on Cancer, IARC, Lyon, France

**Keywords:** Endocrine cancer, Endocrine cancer

## Abstract

Ki-67 assessment is a key step in the diagnosis of neuroendocrine neoplasms (NENs) from all anatomic locations. Several challenges exist related to quantifying the Ki-67 proliferation index due to lack of method standardization and inter-reader variability. The application of digital pathology coupled with machine learning has been shown to be highly accurate and reproducible for the evaluation of Ki-67 in NENs. We systematically reviewed all published studies on the subject of Ki-67 assessment in pancreatic NENs (PanNENs) employing digital image analysis (DIA). The most common advantages of DIA were improvement in the standardization and reliability of Ki-67 evaluation, as well as its speed and practicality, compared to the current gold standard approach of manual counts from captured images, which is cumbersome and time consuming. The main limitations were attributed to higher costs, lack of widespread availability (as of yet), operator qualification and training issues (if it is not done by pathologists), and most importantly, the drawback of image algorithms counting contaminating non-neoplastic cells and other signals like hemosiderin. However, solutions are rapidly developing for all of these challenging issues. A comparative meta-analysis for DIA versus manual counting shows very high concordance (global coefficient of concordance: 0.94, 95% CI: 0.83–0.98) between these two modalities. These findings support the widespread adoption of validated DIA methods for Ki-67 assessment in PanNENs, provided that measures are in place to ensure counting of only tumor cells either by software modifications or education of non-pathologist operators, as well as selection of standard regions of interest for analysis. NENs, being cellular and monotonous neoplasms, are naturally more amenable to Ki-67 assessment. However, lessons of this review may be applicable to other neoplasms where proliferation activity has become an integral part of theranostic evaluation including breast, brain, and hematolymphoid neoplasms.

## Introduction

The world health organization (WHO) released in 2019 a consensus document entitled “Recommendations on digital interventions for health system strengthening”, acknowledging that artificial intelligence (AI) and digital technologies can offer limitless possibilities to advance health management and achievements (https://apps.who.int/iris/handle/10665/311980, last access 10/30/2021). Indeed, AI-based technologies are emerging in every medical field, especially in radiology and pathology^1–3^. In pathology, AI-based systems utilize machine- and/or deep- learning models to assist pathologists in analyzing digital images to perform different tasks, including screening for rare events, quantification, diagnosing lesions, and prognostication^1,4,5^. Digital pathology, which includes the digitizing of glass slides to generate whole slide images, facilitates the application of AI in pathology^6–8^. A key benefit of employing AI-based systems in pathology is to provide reliable, objective and reproducible results, thereby reducing inter- and intra-pathologist variability and enabling automation to augment routine practice^1,6–8^.

**Fig. 1:**
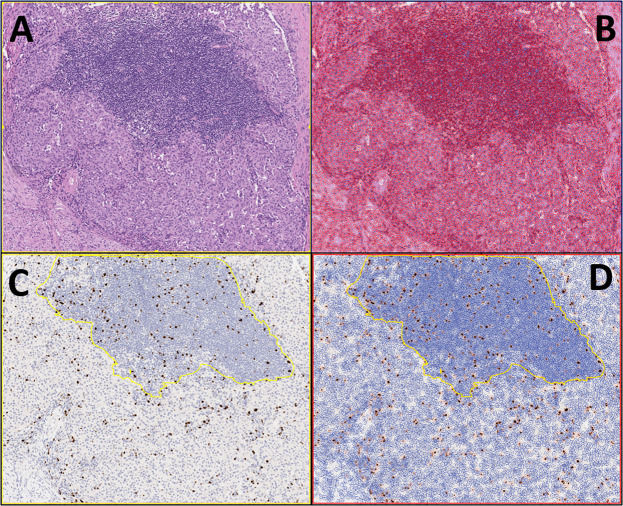
An example of the use of a digitalized system for assessing Ki-67 in pancreatic neuroendocrine neoplasms is shown here. This is a particularly illustrative case due to the presence of a lymphocytic infiltrate at the tumor periphery, which represents a potential source of bias for Ki67 assessment with digital systems. **A** A pancreatic neuroendocrine tumor, G2, is shown. (Hematoxylin-eosin, 10x original magnification); **B** the digitalized system can count all cells present in a specific field, also on hematoxylin-eosin slides; **C**, **D** modern systems can select a specific area for the Ki-67 count: in this example, the field with lymphocytes has been excluded from the count, reducing potential important biases in tumor grading (Ki67 immunohistochemistry, 10x original magnification).

In this context, digital image analysis (DIA) has been utilized to evaluate neuroendocrine neoplasms (NENs). Among well-differentiated neuroendocrine tumors (NETs), grading is based on the assessment of mitotic rate and the proliferation index determined by Ki-67 immunostaining^9–11^. Currently, the WHO classification of some NENs specifies that Ki-67 should be assessed by manual counting on a printed image including at least 500 neoplastic cells from the regions of highest labeling (hotspots)^9,10^. Recently, different DIA-based systems have been developed to assist pathologists with this important task (Fig. 1), which has implications for the clinical management of patients with NENs. To date, the majority of studies on this topic were performed on NENs of the gastro-entero-pancreatic system.

The aim of our study was to systematically review all published studies that compared manual Ki-67 assessment in pancreatic NENs (PanNENs) with DIA-based determination, highlighting the benefits and drawbacks of each approach. A comparative meta-analysis is also undertaken of manual counting versus DIA for PanNENs.

## Materials and methods

This systematic review adhered to the MOOSE guidelines^12^ and PRISMA statement^13^. Studies were considered eligible for inclusion if they reported original data on DIA-based assessment of Ki-67 in PanNENs. Both neuroendocrine tumors (PanNETs) and carcinomas (PanNECs) were included. For the comparative meta-analysis of manual counting vs. DIA, we considered all manuscripts reporting an analytical comparison between these two modalities used in the assessment of Ki-67. In the case of duplicate cohorts, the largest and then most recent was selected. Exclusion criteria included no definitive histological diagnosis of PanNEN, and in vitro or animal studies.

### Data sources and literature search strategy

Two investigators (CL, PA) independently searched PubMed, Embase and SCOPUS databases up until 30/06/2021. The search strategy included combinations of the following keywords: #1 “digital”[Title/Abstract] AND “pathology”[Title/Abstract]; #2 “image”[Title/Abstract] AND “analysis”[Title/Abstract]; #3 “artificial intelligence”[Title/Abstract] OR “AI”[Title/Abstract] OR “machine learning”[Title/Abstract] OR “deep learning”[Title/Abstract] OR “automated”[Title/Abstract] OR “semiautomated”[Title/Abstract] OR “algorithm*“[Title/Abstract] OR “neural network”[Title/Abstract] OR “computer-aid”[Title/Abstract] OR “computer-aided”[Title/Abstract] OR “image analysis”[Title/Abstract] OR “digital pathology”[Title/Abstract] OR “WSI”[Title/Abstract] OR “whole slide”[Title/Abstract] OR “digital”[Title/Abstract]; #4 #1 OR #2 OR #3; #5 “neuroendocrine”[Title/Abstract] OR “carcinoid”[Title/Abstract] OR “medullary”[Title/Abstract]; #6 #4 AND #5; #7 “artificial intelligence”[MeSH Terms]; #8 “Neuroendocrine Tumors”[MeSH Terms] OR “carcinoma, neuroendocrine”[MeSH Terms] OR “Gastro-enteropancreatic neuroendocrine tumor” [Supplementary Concept] OR “Carcinoid Tumor”[MeSH Terms]; #9 #7 AND #8; #10 #6 OR #9.

### Study selection and data extraction

Following the aforementioned search strategy, duplicates were removed and then two reviewers (CL, PA) independently screened titles and abstracts of all potentially eligible articles. These two authors applied eligibility criteria and reviewed the full texts of included studies. A final list of articles was subsequently established for both the systematic review and comparative meta-analysis by consensus with a third independent author (AE). Two authors were involved in extracting data in a preset Excel database: one (CL) extracted data from the selected articles; the other (AS) independently validated the extracted data. For each article, we extracted the following information: authors; year of publication; country study originated from; number of cases; patient demographics; type of analyzed material; tumor grading; as well as methods for manual counting and DIA. For the comparative meta-analysis, the primary outcome was the coefficient of agreement between manual counting vs. DIA in the assessment of Ki-67 in PanNENs.

### Data synthesis, quality, and publication bias assessment

The comparative meta-analysis was conducted using Comprehensive Meta-Analysis v2 software (Biostat; Englewood, NJ, USA). Furthermore, the Newcastle–Ottawa Scale (NOS) was used to assess study quality, following existing guidelines^14,15^. Finally, we investigated publication bias by visual inspection of funnel plots and with the Egger bias test^16^.

## Results

### Search results

The search yielded a total of 4286 potential eligible studies. Following in-depth screening based on title/abstract, only 56 (1.3%) of these studies were retrieved for further analysis. Of them, 22 were considered eligible for the systematic review^17–38^, and 4 for the correlation meta-analysis (Supplementary Fig. 1)^25,27,28,34^.

### Study and patient characteristics

The most important features from the extracted data are summarized in Table 1. Overall, the selected studies reported data on a total of 752 PanNENs. The majority of the investigated cohorts (59.1%) were from the USA, with the remaining composed of European patients (27.3%) and mixed cohorts including Asian patients (13.6%). There was an almost equal distribution of male (50.5%) and female (49.5%) patients. Regarding tumor grading (G), the majority of cases were G1 (55.3%), followed by G2 (40.6%) and G3 (4.1%). The type of specimen material analyzed varied, including surgical resection specimens, biopsies, and cytology cell blocks. The majority of the studies (54.5%) did not report specific data on the type of specimens analyzed. The reported procedures used for manual counting and the specific DIA technologies adopted in the assessment of Ki-67 in PanNENs are summarized in Table 1.

**Table 1:** Summary of studies about AI-based systems used for Ki-67 assessment in PanNENs.

AUTHOR, YEAR	COUNTRY	N° OF CASES	GENDER	MATERIAL	GRADING	MANUAL COUNT	DIA
Bagci, 2012	USA-Japan	21	N/A	SRS	WD	EE, ECM, CC/PI	N/S
Remes, 2012	Finland	31^a^	N/A	N/S	WD	ECM of at least 2000 cells (hotspots)	Publicly available ImmunoRatio software, capturing five different image fields (minimum of 400 tumor cells per picture, altogether 2000 cells)
Fung, 2012	USA	16^b^	N/A	CB	WD	N/S	Automated Cellular Imaging System III (ACIS, Dako, Carpinteria, CA, USA) at 20x objective in 3 tumor “hotspots”
Goodell, 2012	USA	45	22 M, 22F^Ω^	SRS	WD	ECM	VIAS (Ventana): count in 1 hotspot; count in 10 consecutive random fields
Tang, 2012	USA	12^c^	N/A	N/S	WD	1. ECM of >2000 cells 2. EE	Aperio immunohistochemistry nuclear quantitative image analysis (QIA) algorithm analyzing representative images scanned at 20x magnification
Cimic, 2014	USA	28	10 M, 18 F	SRS	WD	EE	Free software available online (Immunoratio.com)
van Velthuysen, 2014	The Netherlands	6^d^	N/A	N/S	N/A	EE at x20	ImageJ freeware at different magnifications (20x and 40x).
Reid, 2015	USA, Turkey, Japan, Korea	68	33 M, 35 F	N/S	26 G1, 39 G2, 3 G3	1. EE at intermediate power (x10 objective) 2. ECM on the x20 objective 3. CC/PI 4. Careful, extensive, and exhaustive analysis by an expert.	Automated cellular image cytometer (ACISs III, Dako) scanned the entire slide at x4 and 3 hotspots were selected
Kroneman, 2015	USA	97	51 M, 46 F	N/S	N/A	1. EE 2. ECM of at least 500 tumor cells	Automated Cellular Imaging System (ACIS) (Dako) to select 8 to 10 hotspots within the hottest staining region(s) of the tumor present on the slide
Mejias, 2015	USA	21	N/A	N/S	7 G1, 14 G2	N/S	Ventana Image-VIAS
Neely, 2016	USA	24	N/A	CB	N/A	CC/PI, selection of 3 hotspots	Calculation of PI on 3 hotspots with a DIA software algorithm
Burdette, 2016	USA	57	N/A	N/S	WD	CC/PI, selection of 6 hotspots	Whole slide scanning with Aperio ImageScope, manual revision and selection of 6 hotspots, Aperio immunohistochemistry nuclear quantitative analysis algorithm
Jin, 2016	USA	58	33 M, 25 F	CB and SRS	31 G1, 23 G2, 4 G3	CC/PI of at least 500 tumor cells. For cases where TTCN was less than 500 on the entire slide, all tumor cells were counted.	Publicly available ImmunoRatio software. Basic mode was used for analysis
Conemans, 2017	The Netherlands	69	N/A	SRS	57 G1, 11 G2, 1 G3	ECM 2000 cells (hotspot)	Digital quantification of Ki67 LI (PACS, Sectra AB, Linköping, Sweden) on manually selected hotspots
Niazi, 2018	USA	33	N/A	Biopsy	WD	N/S	Deep learning method to automatically differentiate between NET and non-tumor regions based on images of Ki67 stained biopsies
Dere, 2019	Turkey	8^e^	N/S	N/S	N/A	ECM of 500 to 2000 tumor cells	Software designed by Technology Faculty of the institution
Sajjan, 2019	USA	50^f^	N/S	N/S	N/A	N/S	Ki67-stained whole slide images were captured and the tumor area with the greatest mitotic activity was manually identified. The Ki67-positive cells were counted in 0.5 mm2 using Ventana Virtuoso software
Owens, 2020	UK	42	N/A	N/S	G1 and G2, NOS	CC/PI, 1 hotspot	Open-source image analysis program QuPath version 0.1.34 analyzing the same hotspot regions used for the manual Ki67 assessments. Each hotspot was classified into tumor and stromal compartments using a detection classifier based on training regions
Saadeh, 2020	Jordan	3^g^	N/S	N/S	WD	CC/PI of at least 1000 tumor cells	ImageJ
Satturwar, 2020	USA	39^h^	N/S	CB	N/A	1. EE 2. CC/PI of up to 3 hotspot at ×20 magnification	Aperio immunohistochemistry color convo-luted, nuclearV9 quantitative image analysis algorithm (Leica Biosystems)
Lea, 2021	Norway	21^i^	N/S	SRS and biopsy	N/A	ECM of 500 to 2000 tumor cells	Visiopharm image analysis software (Hoersholm, Denmark) measured Ki67and PHH3 on IHC slides including 500 to 2000 tumor cells
Boukhar, 2021	USA	3^j^	1 M, 2 F	N/S	2 G2, 1 G3	CC/PI of hotspot images	Two DIA platforms: QuantCenter and HALO
TOTAL	13/22 USA, 6/22 Europe, 3/22 Asia and mixed	752	50.5% M, 49.5% F	12 N/S; 4 SRS, 3 CB, 1 biopsy, 2 other	55.3% G1, 40.6% G2, 4.1% G3, NOS	–	–

Abbreviations: *AI* Artificial intelligence; *MC* Manual count; *DIA* Digital image analysis; *PanNENs* Pancreatic neuroendocrine neoplasms; *CB* Cell blocks; *SRS* Surgical resection specimens; *NET* Neuroendocrine tumor; *N/A* Not available; *EE* Eyeball estimation; *ECM* Eye-counting with microscope; *CC/PI* Camera captured/printed image; *N/S* Not specified; *WD* Well-differentiated; *M* Male; *F* Female; *PI* Proliferation index; *PHH3* Phosphohistone H3; *IHC* Immunohistochemistry, *NOS* Not otherwise specified.

Notes: ^a^This study investigated a total of 51 cases, 31 with pancreatic origin and 20 with ileal origin; ^b^This study investigated a total of 22 cases, 16 with pancreatic origin (including 3 liver metastases) and 6 with gastro-intestinal origin (including 4 liver metastases); ^c^This study investigated a total of 27 cases, 12 with pancreatic origin, 12 originated from small bowel and 3 with rectal origin; ^d^This study investigated a total of 73 cases, 2 with gastric origin, 18 originated from small bowel, 8 with colonic origin, 18 with pulmonary origin, and 6 with pancreatic origin and 21 liver metastases; ^e^This study investigated a total of 50 cases, 26 with gastric origin, 10 with appendiceal origin, 3 with colorectal origin, 3 with ileal origin and 8 with pancreatic origin; ^f^This study investigated a total of 134 cases, 6 with gastric origin, 64 originated from small bowel, 6 originated from large bowl, 7 with appendiceal origin, 31 with mesenterial origin and 50 with pancreatic origin; ^g^This study investigated a total of 20 cases, 3 with pancreatic origin, 2 with gastric origin, 2 with duodenal and ampullary origin, 7 with jejunal and ileal origin, 2 with appendiceal origin and 2 with colonic origin; ^h^This study investigated 50 cases, 39 with pancreatic origin and 11 liver metastases; ^i^This study investigated a total of 159 cases, 2 with esophageal origin, 9 with gastric origin, 54 originated from small bowel, 1 originated form Meckel’s diverticulum, 31 with appendiceal origin, 21 with pancreatic origin, 15 with colonic origin, 14 with rectal origin and 7 liver metastases and metastases with unknown primary tumor; ^j^This study investigated a total of 25 cases, 3 with pancreatic origin, 5 with ileal origin, 5 with duodenal origin, 2 with gastric origin, 3 nodal metastases, 1 ileal metastasis, 5 liver metastases and 1 diaphragmatic metastasis; ^Ω^this study reported data on a total of 45 cases but the total number of patients was 44: there were 22 females (one had two tumors, for a total of 23 tumors) and 22 males.

### Advantages and limitations of DIA-based systems in the assessment of Ki-67

The key advantages and limitations of DIA-based systems in the assessment of Ki-67 in PanNENs are summarized in Table 2. The most commonly described advantages of DIA were improved reproducibility and reliability, as well as reduced time required for Ki-67 assessment. The most common limitations of DIA were counting non-neoplastic (“contaminants”) cells (e.g., lymphocytes), the higher cost compared with manual counting, and the potential delay in diagnosis, which was dependent on some procedures or technician availability.

**Table 2:** Summary of reported advantages and limitations when utilizing DIA systems to assess Ki-67 for PanNENs.

AUTHOR, YEAR	ADVANTAGES	LIMITATIONS
Bagci, 2012	NR	Highest impact on turnaround time, depending on technician availability; low practicality and moderate accuracy
Remes, 2012	Quick, precise and reliable; not influenced by changes in cell size or growth patterns	NR
Goodell, 2012	Efficient method	Can be influenced by counting hotspot vs. randomly selected fields; low reproducibility if standardized thresholds are lacking
Tang, 2012	Ki67 quantification by MC and DIA demonstrate comparable accuracy	Inability to evaluate each tumor cell
Cimic, 2014	Reproducible	NR
van Velthuysen, 2014	Reproducible	NR
Reid, 2015	Pathologist independent	Dependent upon laboratory technician availability and instrument accessibility; high cost
Kroneman, 2015	Almost perfect correlation between MC and DIA	Difficulty with cell counting due to inability to separate individual cells because of indistinct cell borders
Mejias, 2015	NR	Inability to distinguish infiltrating lymphocytes and other non-neoplastic cells
Neely, 2016	Accurate for cytology	Risk of counting non-tumor contaminants (lymphocytes, pigmented macrophages)
Burdette, 2016	Accuracy	NR
Jin, 2016	NR	Non-tumor cell contamination and insufficient sampling
Dere, 2019	Reduction of time for Ki67 evaluation	Expensive
Saadeh, 2020	Accurate, efficient, reliable and reproducible	Inability to evaluate each tumor cell
Satturwar, 2020	Excellent reliability	NR
Lea, 2021	Improved reliability and reproducibility of grading	NR
Boukhar, 2021	Non-inferiority and substantial time savings	Expert morphologic assessment required for quantitative evaluation

Abbreviations: *PanNENs* Pancreatic neuroendocrine neoplasms; *NR* Not reported; *MC* Manual count; *DIA* Digital image analysis.

### Comparative meta-analysis, quality and publication bias assessment

Overall, for the comparative meta-analysis of 4 studies including 238 patients with PanNEN were selected^25,27,28,34^. The pooled correlation estimate was 0.94 (95%CI: 0.83–0.98; I^2^ = 24.15%), indicating a high correlation between manual (reference value) and digital count. The heterogeneity was low (i.e., I^2^ < 50%), reinforcing the reliability of these results. The quality of the studies did not represent risk of bias (mean score of the Newcastle–Ottawa Scale: 8). Furthermore, no publication bias emerged (Egger’s test = 1.42; *p* = 0.90). The fail-safe number was 660, a value that indicates strong statistical significance of our results based on existing guidelines^15,16^.

## Discussion

The Ki-67 proliferative index is critical in the pathologic assessment of PanNEN, and has important clinical implications for patient management. The adoption of international recommendations released by the WHO classification of tumors and the European neuroendocrine tumor society (ENETS) for assessing Ki-67 has improved the standardization of methodologies for this task^9,39^. However, given the persistence of interlaboratory and interobserver discrepancies, as well as potential inconsistencies with different scoring systems, accurately grading PanNENs remains a challenge for pathologists, especially in the G1-G2 and G2-G3 transition areas for PanNETs. Multiple factors affecting the interpretation of the Ki-67 index include the use of different antibody clones and staining protocols, tissue section thickness, tumor cell density, and difficulty distinguishing tumor from non-tumor cells. According to Adsay, “to count or not to count is not the question, but rather how to count”^40^. Manually counting camera captured or printed images is generally favored over eyeballing. Further, more recently DIA has proven to be an acceptable method for Ki-67 assessment. In this study, we reviewed all published investigations that employed DIA for Ki-67 determination in PanNENs, highlighting some of the advantages and limitations of utilizing this technology. Furthermore, by comparing the coefficient of correlation between manual counting and DIA by means of a comparative meta-analysis, we demonstrated a high value of consistency (0.94, 95%CI: 0.83–0.98) between these two approaches.

The advantages derived from utilizing DIA include more reproducible results, higher accuracy, and reduced time to evaluate Ki-67 in PanNENs^1,6–8^. Current guidelines for assessing Ki-67 recommended manual counting from a printed image that includes at least 500 neoplastic cells from tumor hotspots. While still time consuming, this manual method does promote standardization that helps reduce interobserver variability^24^. However, for grade transitions between G1 and G2 (3% of Ki-67) and between G2 and G3 (20% of Ki-67), there were still discrepancies with manual counting from a printed image. The use of DIA for Ki-67 determination resulted in greater consistency in grading of all PanNEN cases, particularly for those cases belonging to the aforementioned gray transition areas G1-G2 and G2-G3. However, it should be noted that even when using DIA one can obtain different results depending on the selection of hotspots and by altering the number of cells counted. Access to DIA allows rapid counting of more cells, and that alone can push a tumor from G2 to G1 or G3 to G2, whereas counting fewer cells in the same hotspot can achieve the reverse^41^.

DIA assistance with grading PanNEN is of particular benefit in fine needle aspiration (FNA) cytology samples. Guidelines established using histological samples have been extrapolated to grading PanNENs in cytology material (e.g., cell blocks) procured by FNA. Several studies have demonstrated that Ki-67 assessment by manual counting of tumor cells in cell blocks can result in under-grading of these neoplasms when compared with matched surgical resection specimens^36,42^, with discrepancies more often observed in G2 cases^20,29^. Intriguingly, Abi-Raad and colleagues demonstrated that counting hotspots instead of the complete cell block can provide a higher concordance with surgical specimens, especially for FNA samples that contain ≥ 1000 cells^43^. A different perspective was provided by Satturwar and colleagues who investigated the potential role of augmented reality microscopy (ARM) for Ki-67 assessment in cytology specimens^36^. ARM, which is basically a modified microscope associated with an attached computer unit, enables real-time image analysis using a traditional light microscope and glass slides, without the need to first photograph or digitize slides^36,44,45^. If coupled with image analysis software, ARM allows quantifying immunohistochemical stains including Ki-67, and can also be combined with elaborate AI-based algorithms to perform more complex tasks^44–46^. Like other DIA methods, ARM has significant potential for improving PanNEN grading on cell block material^36^.

Currently, DIA for Ki-67 assessment has some limitations that may need to be addressed if counting in practice is to be performed with this approach. The most commonly reported drawback is the risk of counting dividing non-neoplastic “contaminating” cells (e.g., endothelial cells, lymphocytes), thereby erroneously increasing the overall tumor grade. Other brown-pigmented signals (hemosiderin and hematoidin) also cause this over-counting phenomenon. Such issues are enhanced in NEC, especially due to the effect of artefacts (e.g., smeared chromatin material, nuclear molding in small cell NEC) on DIA. However, these problems can be overcome by having pathologists directly annotate regions of interest to be scored, with the intent of excluding contaminating cells from being counted. Further studies that specifically address these challenges in PanNEC are needed. This issue becomes particularly important if non-pathologist personnel such as trainees and technicians are used as key operators. Of note, more sophisticated AI-algorithms are being developed that only count neoplastic cells^47–49^ and become more operator-independent. One potential solution that also has been employed is to utilize double-stained slides (e.g., Ki-67 and synaptophysin) with deep learning algorithms to improve the accuracy of Ki-67 index quantification^50–53^. More recently, some investigators have shown that they are able to predict Ki-67 positive cells directly from H&E images using AI-based methods^51^. Another important pending issue that needs to be addressed for improving Ki-67 assessment in PanNENs is related to standardizing hotspot size and number to be evaluated^41,54^. Hotspots are defined as tumor areas with higher Ki-67 nuclear staining. It has been shown that the greater the hotspot size, the lower the Ki-67 count, highlighting the importance of standardizing this parameter for reliable evaluation^34,41^. Furthermore, not only is the size of the hotspot difficult to define, but so is the shape^55^. Most pathologists and algorithms define a hotspot as a circular shape; however, there is no biological evidence to support this notion. Another important factor to consider is the number of hotspots when determining the Ki-67 index. Training operators not to select a geographic region that may lead to hyper-selection of positive cells in a given hotspot region is also important, which erroneously creates higher “percentage” positivity. However, all of the above shortcomings are relatively easy to address with proper training and application of improved AI software.

Despite the clear advantages of DIA for determination of the Ki-67 labeling index, scoring with this digital modality is still subject to the fundamental limitation that applies to any cut-off in a continuous variable: it can be changed randomly, as it was for PanNETs in 2017 when it was moved from 2 to 3 for Grade 1^9^. Moreover, any cut-off of a continuous variable can be shown to have value, but the actual grading is inherently arbitrary^41^. Indeed, how best to employ K-i67 as a reliable prognosticator of PanNETs has been a study in progress. For example, in 2017 the cut-off was clarified such that cases with an index less than 3.0 (including 2.99), which were previously unclear as to which grade this belonged, now clearly included Grade 1^9^. Naturally, as in any grading and staging system that assesses a continuous variable, the Ki-67 index-based system is imperfect^54^. For example, it can be expected that cases with 2.99 (now in G1) and 3.0 (now in G2) will be similar in biological behavior. Nevertheless, DIA will help standardize the process, not only offering more reproducible results in daily clinical practice, but also allowing for better comparison between studies that aim to fine-tune this grading system. For example, there have been proposals to move the G1/G2 cut-off to 5%; but it is difficult to verify the results of these proposals due to variation in counting methods. Fundamentally, the reality is that even with more accurate analysis provided by DIA, a G2 tumor with a Ki67 of 4% will still be more likely to behave in an indolent fashion than a G2 tumor with a Ki67 of 19%. Thus, the issue of a continuous variable, which is a complex concept involving statistical and biological sciences^56^, enhances the need for accurate Ki-67 quantification and may ultimately be more important than the actual grade score. Finally, a significant limitation of DIA for widespread adoption has been the accessibility of this technology due to cost and maintenance. However, as whole slide scanners and digital cameras (and related software) become more widely available, the adaptation of facilities to perform DIA for Ki-67 counting is becoming increasingly feasible and amenable to employ^57^. Another issue to be considered is the need to better integrate Ki-67 counting by DIA into routine workflow^24,58^.

In this review, we chose to focus on PanNENs. However, the topic of manual vs. digital pathology scoring of Ki-67 is also certainly of importance for NENs at many other anatomic sites^54^, as well as for other neoplasms in which DIA-based systems are being leveraged to assess biomarkers. In 2015, Joseph et al. studying a cohort of 48 lung carcinoids, demonstrated an overall similarity of manual counting vs. DIA; although Ki-67 estimation resulted in slightly higher results than manual counting^59^. Of note, a more recent analysis by Swarts et al. comparing the use of manual analysis vs. DIA (in-house Leica Qwin program) in a cohort of 201 lung tumors, described a substantial equivalence of both methodologies^60^. It is also worth noting that Ki-67 assessment may be of importance in other tumor types. For example, in 2020 Hida et al. compared the use of manual analysis vs. DIA (Visiopharm software) for proliferative index evaluation in a total of 413 cases of breast cancer, showing a high value of correlation (coefficient of correlation = 0.82, *p* < 0.0001) between both methods^61^. Alataki et al. corroborated these findings, demonstrating a high correlation in Ki-67 assessment between manual and DIA in both surgical breast resections and biopsies^62^.

An important question is whether the comparison of Ki-67 assessment between manual vs. DIA-based systems influences clinical management and prognostication. Among all selected manuscripts, only four provided data on this specific topic^20,23,25,30^. Goodell et al. demonstrated significant reliability in predicting nodal and distant metastasis of PanNETs with the ventana image analysis system (VIAS), with the highest specificity (94% in their cohort) demonstrated when analyzing 10 consecutive and randomly selected fields^20^. Similarly, van Velthuysen et al. investigating the performance of manual vs. digital (ImageJ) Ki-67 scoring in a cohort of 73 PanNENs, showed that tumor grading correlated with survival irrespective of the way Ki-67 was assessed^23^. Similar results were replicated by Kroneman et al.^25^. and Conemans et al.^30^, showing substantial similarities in terms of prognostication between manual vs. DIA scoring of Ki-67. It is important to note that only four studies in the literature provided data on this fundamental topic. Moreover, all of these studies were conducted prior to the introduction of the 2017 grading system. Thus, further studies on larger cohorts and based on current grading methods are needed. We advocate that DIA-based systems could provide a more standardized method, guaranteeing a more reliable basis for prognostic stratification.

In summary, this systematic review and comparative meta-analysis demonstrates that the advantages outweigh the limitations of using DIA to assess Ki-67 in PanNENs. We advocate that the next logical step for more broadly adopting DIA in pathology practice would be to further explore the relationship between hotspot parameters (number, size, and shape) and the Ki-67 index with patient outcome. Currently, most studies use manual counting from captured images as the gold standard; however, the ultimate validation will naturally come from prognostic correlation. Based upon current evidence provided by peer-reviewed literature, DIA appears to offer pathologists higher reliability and reproducibility than manual counting for grading PanNENs. The overall findings of this review, therefore, support widespread adoption of carefully optimized and validated DIA-based methods for this important diagnostic task in clinical practice. Lessons learned from the application of DIA to the PanNEN model can also be extrapolated to different tumors in other organ systems, such as breast carcinoma in which Ki-67 quantification is increasingly becoming a key driver for patient management.

## Supplementary information

Supplementary Figure 1

## Data Availability

All data/information are available in the manuscript and in the supplementary material.
