# Supplementary figures and images for: Ki-67 assessment of pancreatic neuroendocrine neoplasms: Systematic review and meta-analysis of manual vs. digital pathology scoring
Source: Mod Pathol. 2022 Mar 5;35(6):712–20. doi: 10.1038/s41379-022-01055-1 (PMC9174054; doi:10.1038/s41379-022-01055-1)

**Supplementary Figure 1. PRISMA checklist for this systematic review and meta-analysis.**

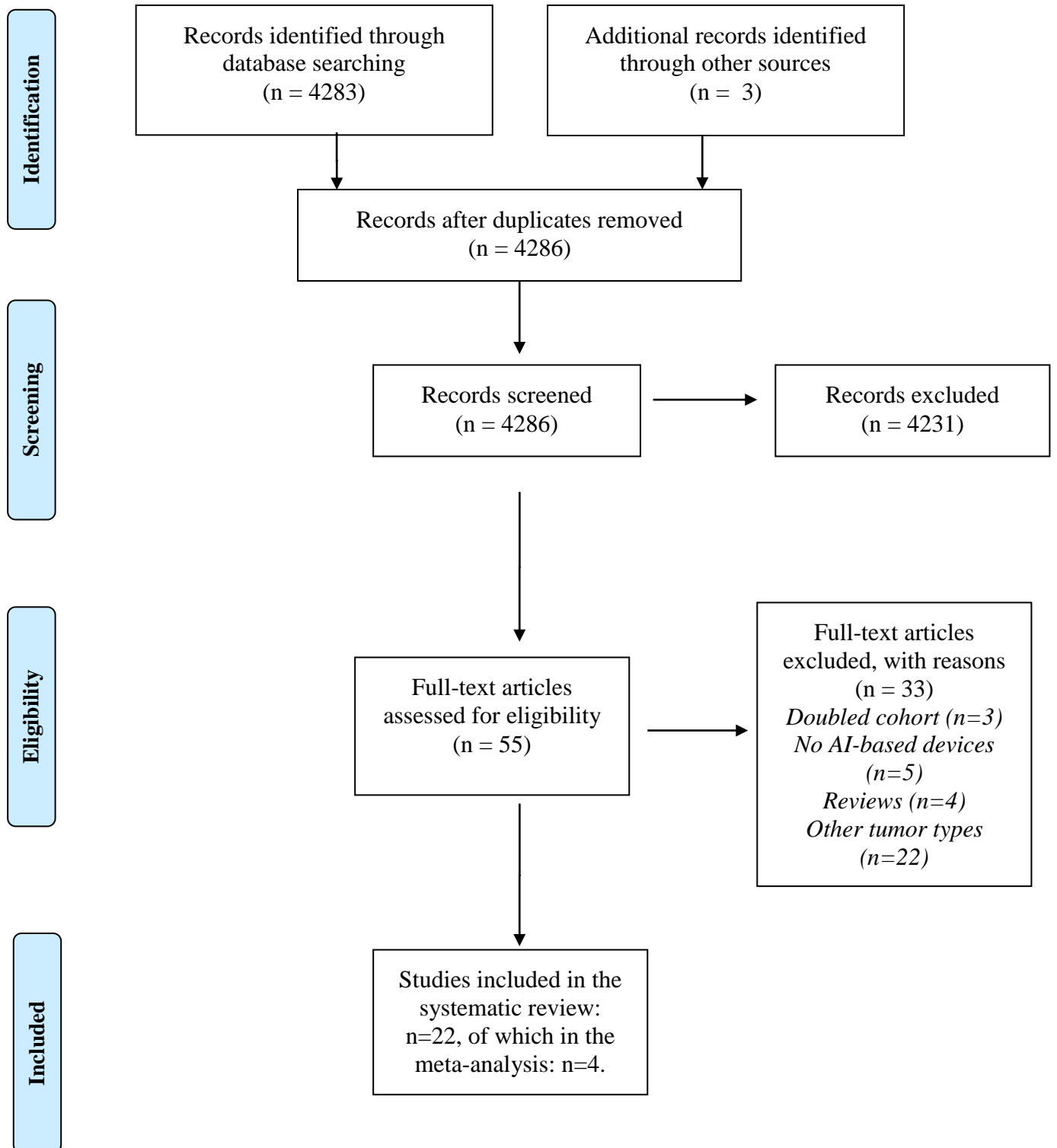

Supplement: Supplementary file 1 — Supplementary Figure 1 [file 41379_2022_1055_MOESM1_ESM.pdf]
